# Supplementary material for: Patterns of Intron Gain and Loss in Fungi
Source: PLoS Biol. 2004 Nov 30;2(12):e422. doi: 10.1371/journal.pbio.0020422 (PMC532390; doi:10.1371/journal.pbio.0020422)
Supplement: Table S1 — Also available at http://genes.mit.edu/NielsenEtAl/. (4.3 MB ZIP). [file pbio.0020422.st001.zip › NielsenEtAl/html/845.html]

AN0051.1.NCU06416.1.MG06487.1.FG07303.1


```
 CLUSTAL W (1.82) Multiple Sequence Alignments - Introns Inserted


Sequence 1: NCU06416.1	333 aa
Sequence 2: MG06487.1	336 aa
Sequence 3: FG07303.1	343 aa
Sequence 4: AN0051.1	346 aa
Alignment Length: 354 aa
Number Identitical Residues: 160 aa
Alignment Score (without introns) 7942


MG06487.1 	------~---MEKAAVSAEGLLIP0LIDFSKFLNGNDAQRQETAKAILHGFQTAGFIYLK
NCU06416.1	------~---MEKAAVNEDGLVIP0LIDFSKFLEGDETLKLETAKAILHGFQTAGFIYLK
FG07303.1 	MTSDLA0VGPYLERTSSYQLIHLK~LIDFSLFLNGTPSERNDTANAILEGFKTAGFIYLK
AN0051.1  	--MPVQ~ACYPITETVSKDGLVIP0IINFAPFRNGTPADKHAVAMSVVHAFKTSGFLYLK
          	              : . : : :  :*:*: * :*  : :  .* :::..*:*:**:***

MG06487.1 	NLPLEPEVLSHVFATSAKFFTVPLETKMDLAWTTPASNRGYSAPGREKVTQLADIDEVKK
NCU06416.1	NIPIQPDFREHVFNTSAKFFKLPKEKKLEVGWTTPEANRGYSAPGREKVTQLTDPAEIEK
FG07303.1 	NHPVSPKELQHAFDMSARWFDQPLESKMKVVWTTPEANRGYSAPGREKVSQLTDLADVEK
AN0051.1  	NHGIPPSVTSRVFKSSASFFGRPQDQKDTLGWTTPQANRGYVRTGREKLSTVDDQADRT-
          	*  : *.  .:.*  ** :*  * : *  : **** :****  .****:: : *  :   

MG06487.1 	LKDGNPDMKESFEIGREGVPEYPNQWPKDDTDATSEFKPTMLDFFDRCKAIHVEVMRAIA
NCU06416.1	IRSAAPDIKESYEIGREDEPGHPNPWPAEQDDLVG-FKSTMNNFFDQCKALHIEVMRAIA
FG07303.1 	MRAALPDIKESLEIGRENEAGHPNYWLEESGELVG-FRKDMLGFFEQCRQLHVEVMKAIA
AN0051.1  	----VPDIKESMEIGREGVEGLPNRWPDQLDEEGKDFKNVMLEFFEMCKELHIKVMQAIA
          	     **:*** *****.    ** *  :  :   .*:  *  **: *: :*::**:***

MG06487.1 	VGMGLEDPGYFDRFVDVGDNTLRLLHYPAVDRNVFQINPGQVRAGEHS~DYGSITLLFQD
NCU06416.1	VGMGIDAN-YFDSFVDVGDNILRLLHYPAVKSEVFKINPGQVRAGEHT~DYGSITLLFQD
FG07303.1 	VGMGLDDG-FFDGFVDVGDNTLRLLHYPSVQSNVFKMNPGTVRAGEHS0DYGSITLLFQD
AN0051.1  	LGMNLPVH-FFDEYVDKGDNNLRLLHYPAVHKDVFKKNPGQVRAAEHS~DYGSITLLFQD
          	:**.:    :** :** *** *******:*. :**: *** ***.**: ***********

MG06487.1 	ARGGLQVKGPNGAFVDATPIPGTCVVNAGDLLARWSNDTIKSTIHRVVEPP------SGE
NCU06416.1	SRGGLQVKSPNGQFIDATPIENTVVVNAGDLLARWSNDTIKSTVHRVVEPP------KQE
FG07303.1 	VRGGLQVKSPTGQFVDATPIEGTVVVNAGDLLARWSNDMIKSTIHRVVEPP------QKE
AN0051.1  	RRGGLQARSPEGTWVDVTPFPDTVVINAGDLLARWSNDTIKSTKHRVIEPPKVPGAEDDD
          	 *****.:.* * ::*.**: .* *:************ **** ***:***. ..:.. :

MG06487.1 	GDVYPPRYSIA2YFCNPNSDAYIETIPGTYQTE---ADKKYEGINSGKYLEQRLAATY
NCU06416.1	-DVHPPRYSIA2YFCNPNHKSYIEAIPGTYAAE---SERKYEGINSGKYLVQRLAATY
FG07303.1 	AESYPPRYSIA2YFCNPNFKSFIEAIPGTFETE---KDKKYDGINSGDYLVQRLTATY
AN0051.1  	TDMYPERYSVA~YFCNPNMDKFIEAIPGTYGEGEGLSKAKYPGITSGDYLVQRLSATY
          	 : :* ***:* ****** . :**:****:   ..  . ** **.**.** ***:***
```
